# Inhibition of Proteasome Activity Upregulates IL-6 Expression in RPE Cells through the Activation of P38 MAPKs

**DOI:** 10.1155/2018/5392432

**Published:** 2018-07-12

**Authors:** Tingyu Qin, Shasha Gao

**Affiliations:** Department of Ophthalmology, The First Affiliated Hospital of Zhengzhou University, Zhengzhou 450000, China

## Abstract

**Purpose:**

As far as we know, during the development of age-related macular degeneration (AMD), the activity of proteasome in retinal pigment epithelium cells (RPE) gradually decreases. And a lot of research has shown that age-related macular degeneration is closely related to inflammation and autoimmune. Moreover, there are many cytokines (CKs) involved in the course of inflammation. In this study, we are going to investigate how the decrease of proteasome activity affects the production of interleukin-6 (IL-6) in human retinal pigment epithelium cells (ARPE-19).

**Methods:**

Cultured ARPE-19 was treated with or without MG132, a proteasome inhibitor, and the levels of IL-6 mRNA (messenger ribonucleic acid) in RPE at 1 h, 4 h, 8 h, and IL-6 protein in the culture medium at 2 h, 4 h, 6 h, 8 h, 10 h, and 12 h were measured by real-time polymerase chain reaction (real-time PCR) and enzyme-linked immunosorbent assay (ELISA). The protein levels of MCP-1 (monocyte chemoattractant protein-1) in the culture medium at 2 h, 4 h, 6 h, 8 h, 10 h, and 12 h were also measured by ELISA. Then we tested which of cell signal pathways regulating the production of IL-6 were activated when we added MG132 into the medium by Western blot and electrophoretic mobility shift assays (EMSA). After that, we put the inhibitors of these activated cell signal pathways into the medium individually to see which inhibitor can counteract the effect of upregulating the levels of IL-6 in the culture medium of RPE.

**Results:**

MG132 decreased the secretion of MCP-1 in the culture medium of RPE, but it increased the expression of IL-6 mRNA in RPE and IL-6 protein level in the culture medium of RPE. MG132 treatment was also found to enhance the level of phosphorylated p38 mitogen-activated protein kinases (MAPKs) and c-Jun N-terminal Kinase (JNK) by Western blotting. More importantly, the effect of MG132 on upregulating the levels of IL-6 was inhibited by SB203580, an inhibitor of P38 MAP kinases. But the JNK inhibitor, SP600125, cannot prevent the effect of upregulating the levels of IL-6 by MG132 in the RPE culture medium.

**Conclusions:**

We concluded that the proteasome inhibitor, MG132, upregulates IL-6 production in RPE cells through the activation of P38 MAPKs.

## 1. Introduction

Age-related macular degeneration (AMD) is a disease that causes varying degrees of blindness in senior people especially in developed nations [1, 2], and the mechanism of this disease is still unclear. Considerable evidence shows that retinal oxidative stress [3] and inflammation [4] have been documented with strong association with the development of AMD, both of which are partially regulated by the ubiquitin-proteasome pathway (UPP) [5–7].

The UPP is the chief nonlysosomal proteolytic pathway and protein quality control system within cells and has been implicated in many cellular processes [8–12], including regulation of inflammation and immune reaction [13, 14]. Impairment of the UPP has been involved in the pathogenesis of many age-related degenerative diseases, such as Parkinson's disease [15], Alzheimer's disease [16], diabetic retinopathy [17], senile cataract [18–21], and AMD [22].

There has been growing evidence indicating that development of AMD is related to dysfunction of retinal pigment epithelial (RPE) cells [23–25] and the inflammation is an important component of AMD [4, 24, 26]. Moreover, the oxidative stress in RPE can induce the activation of the complement system [27], which can increase the expression of IL-6, an important proinflammatory cytokine [28–30]. As other kinds of cells, RPE have a normally active UPP [17], and the activity of UPP decreases in different human tissues (including skin, muscle, kidney, liver, lung, heart, lentis, and RPE) with the increase of age [17, 31, 32], however, the relationship between the decline in proteasome activity in RPE and the production of inflammatory cytokine IL-6 which plays an important role in cell growth and inflammatory reactions [30, 33] remains to be obscured.

To further investigate the relationship between the inactivity of proteasome and the expression of IL-6 in RPE, we evaluated the effect of inhibition of proteasome activity on the production of IL-6 as well as other relevant inflammatory cytokines and its mechanism. The data suggest that the inactivity of proteasome upregulates the IL-6 expression in RPE cells through the activation of the P38 MAPKs pathway.

## 2. Materials and Methods

### 2.1. Materials

All materials used for sodium dodecyl sulfate polyacrylamide gel electrophoresis (SDS-PAGE) were purchased from Bio-Rad Laboratories (Hercules, CA). The DuoSet enzyme-linked immunosorbent assay (ELISA) kits for IL-6 and MCP-1 were purchased from R&D Systems (Minneapolis, MN). Fetal bovine serum (FBS), Dulbecco's modified Eagle's medium (DMEM), and antibiotics for cell cultures were purchased from Invitrogen (Carlsbad, CA). MG132 (proteasome inhibitor) and SP600125 (c-Jun N-terminal kinase inhibitor, JNK inhibitor) were purchased from Calbiochem (La Jolla, CA). SB203580 (p38 MAPKs inhibitor) and the monoclonal antibody against *β*-actin were purchased from Sigma. Rabbit polyclonal antibodies against phosphorylated p38 MAPKs and total p38 MAPKs and rabbit monoclonal antibodies against phosphorylated c-Jun and total c-Jun were purchased from Cell Signaling Technology (Danvers, MA). The SuperSignal chemiluminescent detection kit was purchased from Pierce. The AP-1 oligonucleotide was purchased from Promega (Madison, WI). RNeasy mini kit (50) was purchased from QIAGEN. SuperScript III Reverse Transcriptase was bought from Invitrogen (Carlsbad, CA).

### 2.2. Cell Culture and Treatments

ARPE-19 (the human RPE cell line) [34] was obtained from ATCC. The cells were cultured in DMEM in addition with 10% FBS that containing 100 U/ml penicillin G and 100 *μ*g/ml streptomycin and incubated in 5% CO_2_ atmosphere at 37°C. The experiments were run by use of confluent ARPE-19. MG132 was prepared in DMSO at 10 mM and diluted to 10 *μ*M in the cell medium instantly before use. Cells were cultured with MG132 for different time periods as manifested in the figure legends. SB203580 was prepared in DMSO at 10 mM and diluted to 10 *μ*M in the cell medium instantly before use. SP600125 was prepared in DMSO at 50 mM and then diluted to 10, 20, and 40 *μ*M in the cell medium instantly before use.

### 2.3. Western Blot Analysis

ARPE-19 cells were washed twice with cold phosphate-buffered saline supplemented with 2 mM sodium orthovanadate (a phosphatase inhibitor) and instantly collected in SDS loading buffer. Then cell lysates were denatured at 100°C for 5 min. The assay for protein quantification is the BCA method. Equal amounts of protein were resolved on 10%–12% SDS-polyacrylamide gels and transferred to nitrocellulose membranes. Membranes were probed with rabbit polyclonal antibodies against phosphorylated p38 MAPK, total p38 MAPK, phosphorylated JNK and total JNK, rabbit monoclonal antibodies against phosphorylated c-Jun and total c-Jun, or mouse monoclonal antibody against *β*-actin. After cultivation with the corresponding horseradish peroxidase-conjugated secondary antibodies, the specific bound antibody was visualized by use of SuperSignal chemiluminescent detection kit.

### 2.4. Enzyme-Linked Immunosorbent Assay (ELISA)

Levels of IL-6 and MCP-1 secreted into the medium by RPE were determined by ELISA. The medium was diluted 3 times for determining il-6 levels and 6 times for determining levels of MCP-1. Each ELISA was performed in the light of the manufacturer's instructions.

### 2.5. Real-Time PCR and Reverse Transcription PCR (RT-PCR)

Total RNA was obtained from cells by use of the RNeasy mini kit. 2 micrograms of total RNA from each sample were used for reverse transcription by SuperScript First-Strand cDNA Synthesis System for RT-PCR. The sequences of IL-6 and GAPDH used for real-time RT-PCR are 5′-AATAACCACCCCTGACCCAAC (Forward primer), 5′-ACATTTGCCGAAGAGCCCT (Reverse primer) and 5′-TCACCATCTTCCAGGAGCGA-3′ (Forward primer), 5′-CTTCTCCATGGTGGTGAAGAC-3′ (Reverse primer). Real-time RT-PCR analysis was conducted on Stratagene Mx4000 multiplex quantitative PCR system using SYBR Green PCR master mix (Qiagen) based on the manufacturer's instructions. The expression levels of the genes were normalized using GAPDH as a housekeeping gene. The values were calculated using the 2^−ΔΔCt^ method.

### 2.6. Electrophoretic Mobility Shift Assays (EMSAs)

EMSAs were used to determine the DNA-binding activity of AP-1 in the nuclear extracts. Briefly, equal amounts of nuclear extract (2 mg protein) were incubated for 20 min at RT with 10 fmol of 32P-labeled oligonucleotide (1 × 10^5^ cpm) specific for AP-1 in 20 ml binding buffer (50 mM NaCl, 10 mM Tris-HCl, pH 7.5, 1 mM MgCl_2_, 1 mM EDTA, 1 mM DTT, 4% glycerol, 1 mg poly(dI-dC), and 1 mg BSA). The DNA-protein complexes formed were resolved on 5% nondenaturing polyacrylamide gels using 0.5 × TBE (45 mM Tris-borate and 1 mM EDTA). Autoradiography was performed to visualize the shifted DNA-protein complexes. The Ap-1 DNA-binding double-stranded oligonucleotide used was 5′-CGC TTG ATG AGT CAG CCG GAA-3′ (Promega, Madison, WI). Specific DNA-binding complexes of AP-1 were identified as the band that disappeared when 50-fold excess of cold oligonucleotide competitor was added in binding assays.

### 2.7. Statistical Analyses

Statistical analysis was performed using Student's *t*-test for comparison between the two groups and using one way ANOVA for multiple comparison.

## 3. Results

**Figure 1:**
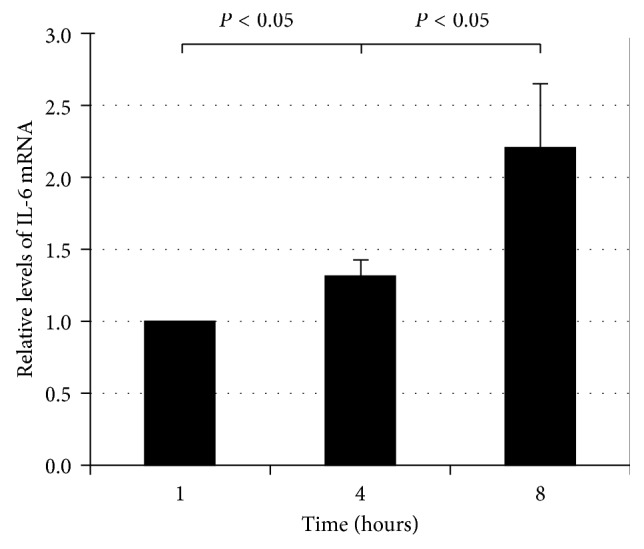
Proteasome inhibition upregulated IL-6 mRNA production in RPE. ARPE-19 cells were cultured in the presence of MG132 for 1, 4, and 8 h. Levels of mRNA for IL-6 were assessed by real-time RT-PCR analysis. GAPDH mRNA was used as a control to normalize the total mRNA levels. The results are the mean ± SD of two independent experiments.

**Figure 2:**
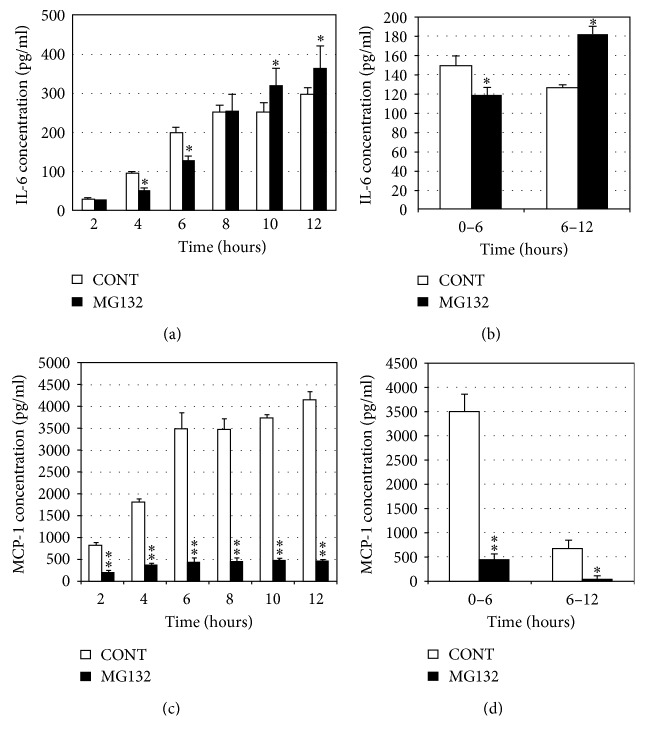
Protease inhibitor MG132 upregulated the protein level of IL-6 in the medium of RPE in MG132 group compared with that in the control group, but it reduced the secretion of MCP-1. (a) Levels of IL-6 in the medium were detected by ELISA following incubation of ARPE-19 cells in the presence or absence of MG132 (10 *μ*M) for 2, 4, 6, 8, 10, or 12 h. (b) Levels of IL-6 in the medium were detected by ELISA during 0 h–6 h or 6 h–12 h in the presence or absence of MG132 (10 *μ*M). (c) Levels of MCP-1 in the medium were detected by ELISA following incubation of ARPE-19 cells in the presence or absence of MG132 (10 *μ*M) for 2, 4, 6, 8, 10, or 12 h. (d) Levels of MCP-1 in the medium were detected by ELISA during 0 h–6 h or 6 h–12 h in the presence or absence of MG132 (10 *μ*M). The results are the mean ± SD ^*∗*^*p* < 0.05 and ^*∗∗*^*p* < 0.01 as compared with the control.

The decrease of proteasome activity upregulated the mRNA and protein level of IL-6 in the hRPE compared with that in control group without proteasome inhibition, but it reduces the secretion of MCP-1. We know that AMD is related to the inflammation [29]; therefore, we were wondering if there were some relationships between the decline in proteasome activity and the inflammation. The data (Figure 1) suggested that proteasome inhibition can increase the level of IL-6 mRNA in RPE in a time-dependent manner. Then, we tested the level of IL-6 and MCP-1 in the medium of RPE and found that proteasome inhibitor, MG132 (10 *μ*M), increased the secretion of IL-6 (Figure 2(a)) but reduced the secretion of MCP-1 among 12 hours (Figures 2(c) and 2(d)). Especially, MG132 obviously upregulated the protein level of IL-6 in the RPE compared with that in control group after 6 h (Figure 2(a)). We also found that the increased level of IL-6 in MG132 group was less than that in control group over a period of 0–6 hours, but during the time interval of 6–12 hours, the level of IL-6 in MG132 group was more than that in control group (Figure 2(b)). However, the level of MCP-1 in the medium of RPE in MG132 group was less than that in the control group during 6 hours, and from 6 h to 12 h, the secretion of MCP-1 in MG132 group was also less than that in control group (Figure 2(d)). From these results, we got that the secretion of MCP-1 was different from IL-6 from 6 h to 12 h. The question was that why proteasome inhibition did not inhibit the secretion of IL-6 like MCP-1.

**Figure 3:**
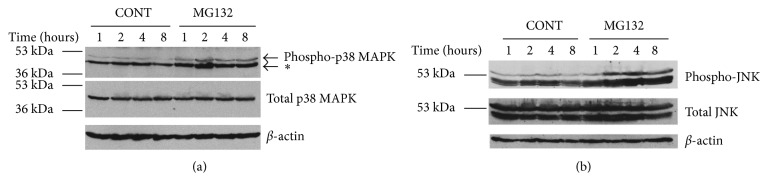
Proteasome inhibitor (MG132, 10 *μ*M) can activate p38 MAPK and JNK in RPE. (a) ARPE-19 cells were cultured in the absence or presence of the proteasome inhibitor MG132 (10 *μ*M) for 1, 2, 4, and 8 h. Levels of endogenous phospho-p38 MAPK, total p38 MAPK, and actin were detected by Western blot using polyclonal (to phosphorylated and total p38 MAPK) and monoclonal antibodies (to actin). ^*∗*^Is the only one phosphorylated site of P38 MAPK. (b) ARPE-19 cells were cultured in the absence or presence of the proteasome inhibitor MG132 (10 *μ*M) for 1, 2, 4, and 8 h. Levels of endogenous phospho-JNK, total JNK, and actin were detected by Western blot using polyclonal (to phosphorylated and total JNK) and monoclonal antibodies (to actin).

**Figure 4:**
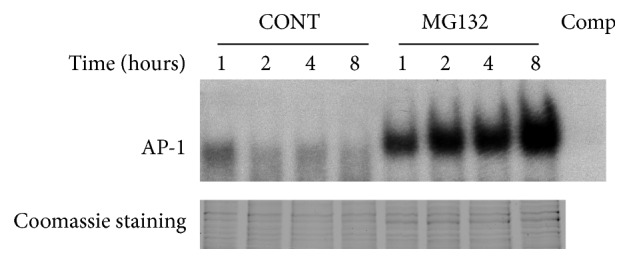
Proteasome inhibition can activate AP-1.

Proteasome inhibition can activate p38 MAPK, JNK, and AP-1. It is known that proteasome inhibition can inactivate NF-*κ*b [22] and the expression of MCP-1 is mainly under the control of NF-*κ*b [35, 36]; therefore, proteasome inhibitor MG132 can decrease the level of MCP-1 in the medium of RPE. But IL-6 is under the control of several cell signal pathways, such as NF-*κ*b, MAPKs, JNK, AP-1, and so on [37, 38]; therefore, to clarify why the secretion of IL-6 was different from MCP-1 when the proteasome activity had been inhibited by MG132, we tested the cell signal pathways, p38 MAPK and JNK. As shown in Figure 3, phosphorylated p38 MAPK and JNK were barely detectable in control cells. Short-term inhibition of the proteasome had no detectable effect on p38 MAPK and JNK phosphorylation; however, inhibiting the proteasome for 2 h or longer resulted in the accumulation of phosphorylated p38 MAPK and JNK. Since JNK and AP-1 both contain Jun [39], and JNK was activated by proteasome inhibition, we investigated the effect of proteasome inhibition on AP-1 activation. In untreated cells, AP-1 was barely detectable; however, treatment with MG132 resulted in a time-dependent phosphorylation of AP-1 (Figure 4).

To study the effect of proteasome inhibition on AP-1 activation, the RPE cells were cultured in the presence or absence of MG132 (10 *μ*M) for 1, 2, 4, and 8 h, nuclear extracts were prepared, and electrophoretic mobility gel shift assays were performed for AP-1 binding. Coomassie staining gel was the internal standard.

**Figure 5:**
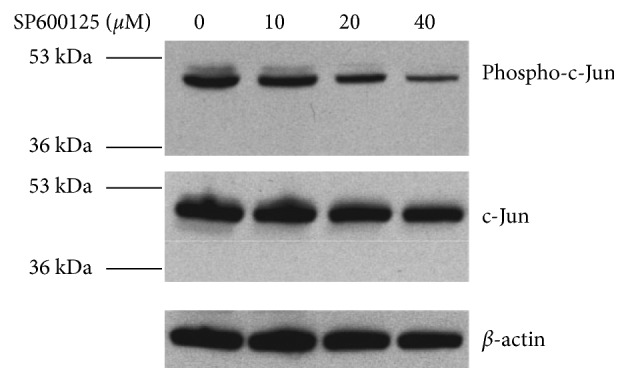
JNK inhibitor (SP600125) can reduce the activation of c-Jun in a dose-dependent way.

**Figure 6:**
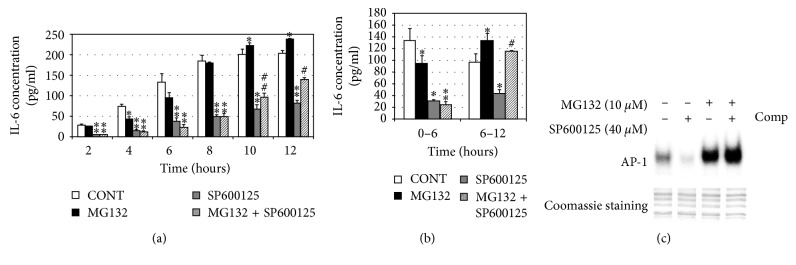
Inhibition of JNK cannot block proteasome inhibition-induced upregulation of IL-6. (a) ARPE-19 cells were cultured in the presence or absence of MG132 (10 *μ*M), Sp600125 (40 *μ*M), or MG132 plus Sp600125 for 2, 4, 6, 8, 10, and 12 h. Levels of IL-6 in the media were determined by ELISA. The results are the mean ± SD. ^*∗*^*p* < 0.05 and ^*∗∗*^*p* < 0.01 as compared with the control; ^#^*p* < 0.01 as compared with the control or Sp600125 alone; ^##^*p* < 0.05 as compared with the control or Sp600125 alone. (b) Levels of IL-6 in the medium were detected by ELISA at 0 h–6 h and 6 h–12 h in the presence or absence of MG132 (10 *μ*M), SP600125 (40 *μ*M), or MG132 plus SP600125. The results are the mean ± SD. ^*∗*^*p* < 0.05 and ^*∗∗*^*p* < 0.01 as compared with the control; ^#^*p* < 0.05 as compared with the control or Sp600125 alone. (c) To study whether JNK inhibition can block the activation of AP-1 induced by proteasome inhibition, the RPE cells were cultured in the presence or absence of MG132 (10 *μ*M), SP600125 (40 *μ*M), or MG132 plus SP600125 for 4 h; nuclear extracts were prepared; and electrophoretic mobility gel shift assays were performed for AP-1 binding. Coomassie staining gel was the internal standard.

Proteasome inhibition upregulating the concentration of IL-6 compared with the control group was not through activating JNK in the RPE. The data above suggest that JNK should be an important signal pathway to regulate the secretion of IL-6. To study whether the upregulation of IL-6 by proteasome inhibition was done through activating JNK or not, we added JNK inhibitor (SP600125) in the medium of RPE. First, we tested what dose of SP600125 was effective to inhibit the phosphorylation of c-Jun. As shown in Figure 5, 40 *μ*M SP600125 was the most effective one, so we added 40 *μ*M SP600125 in the medium of RPE. We found that JNK inhibitor can reduce the basic level of IL-6 in SP600125 group compared with the control group (Figure 6(a)), but it cannot counteract the effect of MG132. During 6 h, there was no obvious difference between SP600125 group and SP600125 plus MG132 group in the level of IL-6 secreted by the RPE, but from 6 h to 12 h, the production of IL-6 by RPE in the SP600125 plus MG132 group was much more than that in the SP600125 group or the control group (Figure 6(b)). To make sure why SP600125 can decrease the basic level of IL-6 in the medium of RPE but it cannot inhibit the upregulation of IL-6 by proteasome inhibition, we tested the effect of SP600125 on AP-1 with or without proteasome inhibition. As shown in Figure 6(c), we can see that JNK inhibitor can reduce the AP-1 DNA-binding activity without proteasome inhibition at 4 h, but it cannot reduce AP-1 DNA-binding activity with proteasome inhibition. From these results, we can explain that JNK inhibition can decrease the basic level of IL-6 which is partly regulated by AP-1 [38] but cannot thoroughly explain why SP600125 cannot decrease the upregulation of IL-6 by MG132 after 6 h. Maybe, the upregulation of IL-6 by proteasome inhibition is related to the activation of AP-1 or not.

ARPE-19 cells were cultured in the absence or presence of the JNK inhibitor (SP600125: 10, 20, 40 *μ*M) for 4 h. Levels of endogenous phospho-c-Jun, total c-Jun., and actin were detected by Western blot using monoclonal (to phosphorylated and total c-Jun) and monoclonal antibodies (to actin).

**Figure 7:**
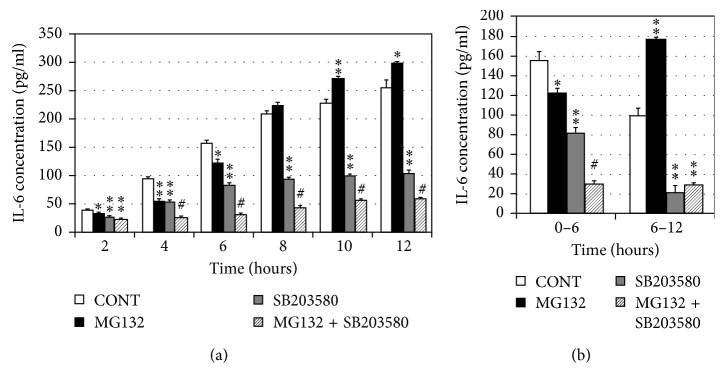
Inhibition of p38 MAPK blocks proteasome inhibition-induced upregulation of IL-6. (a) ARPE-19 cells were cultured in the presence or absence of MG132 (10 *μ*M), SB203580 (10 *μ*M), or MG132 plus SB203580 for 2, 4, 6, 8, 10, and 12 h. Levels of IL-6 in the media were determined by ELISA. The results are the mean ± SD. ^*∗*^*p* < 0.05 and ^*∗∗*^*p* < 0.01 as compared with the control; ^#^*p* < 0.05 as compared with the control or SB203580 alone. (b) Levels of IL-6 in the medium were detected by ELISA at 0 h–6 h and 6 h–12 h in the presence or absence of MG132 (10 *μ*M), SB203580 (10 *μ*M), or MG132 plus SB203580. The results are the mean ± SD. ^*∗*^*p* < 0.05 and ^*∗∗*^*p* < 0.01 as compared with the control; ^#^*p* < 0.05 as compared with the control or SB203580 alone.

Proteasome inhibition upregulating the concentration of IL-6 compared with the control group was through activating P38 MAPKs in the RPE. The data above suggested that P38 MAPKs should be also an important signal pathway to regulate the secretion of IL-6 when we block the proteasome activity of RPE. Therefore, we added the P38 MAPKs inhibitor, SB203580 10 *μ*M according to Fernandes et al. [22], into the DMEM to culture the RPE. As shown in Figure 7(a), P38 MAPKs inhibitor can not only reduce the basic level of IL-6 in SB203580 group compared with the control group, but also it can counteract the effect of MG132; that is to say, the inactivation of P38 MAPKs can inhibit the upregulation of IL-6 by proteasome inhibition. We also found that there was no difference between SB203580 group and SB203580 plus MG132 group in the level of IL-6 secreted by the RPE during the period of 6 h to 12 h (Figure 7(b)). Combing together the results above, we got that proteasome inhibition making the production of IL-6 in the medium of RPE achieve the similar level with the control group was done through activating P38 MAPKs. Next, we want to know whether P38 MAPKs activated by proteasome inhibition will activate AP-1.

**Figure 8:**
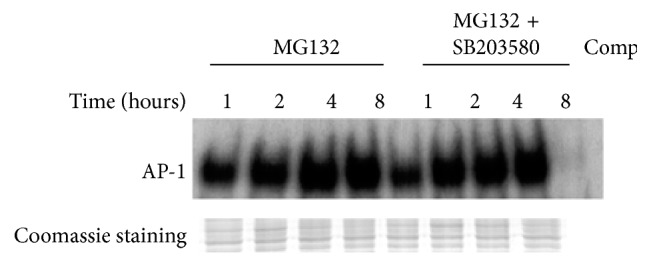
The activation of AP-1 induced by proteasome inhibition is not blocked by the inactivation of P38 MAPK.

P38 MAPKs inhibition cannot block the activation of AP-1 induced by proteasome inhibition. To research if P38 MAPKs inhibition will block the activation of AP-1 induced by proteasome inhibition, we cultured the RPE in the presence of MG132 (10 *μ*M) and in the presence or absence of SB203580 (10 *μ*M) at 1-, 2-, 4-, and 8-hour time point (Figure 8). Then we found that there were no obvious differences between MG132 group and MG132 plus SB203580 group in the AP-1 DNA-binding activity. Therefore, we did not think P38 MAPKs activated by proteasome inhibition would activate AP-1. But we are pretty sure that the upregulation of IL-6 by proteasome inhibition was through activating P38 MAPKs in the RPE.

To study whether P38 MAPK inhibition can block the activation of AP-1 induced by proteasome inhibition, the RPE cells were cultured in the presence of MG132 (10 *μ*M) and in the presence or absence of SB203580 (10 *μ*M) for 1, 2, 4, 8 h; nuclear extracts were prepared; and electrophoretic mobility gel shift assays were performed for AP-1 binding. Coomassie staining gel as the internal standard.

## 4. Discussion

It was reported that the serum levels of IL-6 correlate with the development of AMD and high serum IL-6 levels were associated with the geographic atrophy type of AMD [29, 40, 41]. In addition, aqueous humor IL-6 level is correlated with the size and activity of CNV in AMD patients, indicating that IL-6 level may be related to the progression of CNV [42].

The new proof shows that the decline of proteasome activity plays an important part in the pathogenesis of AMD [31, 32, 43] and in, AMD the proteasome activity of RPE is impaired and reduced [32, 44]. Proteasome inhibitor (MG132) can enhance the expression of IL-6 in human umbilical vein endothelial cells [45]. But the relationship between MG132 and IL-6 in RPE remains elusive. Moreover, what are the molecular mechanisms that proteasome inhibition can change the secretion of IL-6 in RPE?

We used MG132 to inhibit the proteasome activity of hRPE which can mimic the aging in vitro. We found that MG132 can decrease the level of IL-6 in the medium of RPE in the early period which was not more than 6 h, and it can increase the production of IL-6 in the later period which was more than 6 h. We also found that the situation of MCP-1 secretion was different from IL-6, and MG132 can reduce the level of MCP-1 in the medium of RPE in the whole period.

The UPP is involved in regulating a number of signal transduction pathways [22, 46]. We think that the differences between IL-6 and MCP-1 were resulted from proteasome inhibition changing the cell signal pathways. As everyone knows that the expression of MCP-1 is mainly under the control of NF-*κ*B [38], and the expression of IL-6 is under the control of some cell signal pathways such as NF-*κ*B, MAPKs, AP-1, and so on [29, 37, 38, 47]. Because the MCP-1 and IL-6 are both regulated by NF-*κ*B, and in the previous study, some people used the Bay117082 to inhibit NF-*κ*B and found that Bay117082 can decrease the level of IL-6 and MCP-1 compared with the control during 12 h [22]. The secretion of MCP-1 in MG132 group was similar with the Bay117082 group that is because MG132 can also inhibit NF-*κ*B [22]. But the secretion of IL-6 in MG132 group was different from the secretion of MCP-1, and we guessed that maybe MG132 can activate other cell signal pathways to upregulate the production of IL-6 compared with the control.

Next, we tested some cell signal pathways related to IL-6, and we got that proteasome inhibition can activate p38 MAPK, JNK, and AP-1. Thereby, we added JNK inhibitor (SP600125, 40 *μ*M) or p38 MAPK inhibitor (SB203580 10 *μ*M) to the medium of RPE, then we tested the level of IL-6, and we found that SP600125 can inhibit the basic level of IL-6 without adding MG132 in the medium, but SP600125 cannot suppress the effect of proteasome inhibition. We also found that SB203580 can inhibit the effect of proteasome inhibition. It can decrease the level of IL-6 in the medium of RPE with or without adding MG132. Therefore, we got that proteasome inhibition can upregulate the production of IL-6 through activating p38 MAPK but not JNK. Because AP-1 is the downstream signaling of p38 MAPK and JNK cell signal pathways [39, 48–50], and we tested the phosphorylated level of AP-1 when we added SP600125 or SB203580 and found that SP600125 can decrease the phosphorylated level of AP-1. But SB203580 cannot decrease the phosphorylated level of AP-1 activated by MG132. Therefore, we did not think that proteasome inhibition can upregulate the production of IL-6 compared with the control through activating AP-1. Finally, we concluded that proteasome inhibition can upregulate the production of IL-6 through activating p38 MAPK, but the downstream signal pathway of MAPK p38 still needs further research.

Recently, research on the understanding of IL-6 function has led to the improvement in the treatment of immune-related diseases using novel anti-IL-6 drugs [51, 52]. The future perhaps lies in the development of orally active small molecules that inhibit specific inflammatory signaling pathways; for example, the inhibitor of p38 and its downstream effectors have been proven to be effective in the treatment of autoimmune arthritis in a rat model and have been recently tested in clinical trials [53, 54]. These novel therapeutic strategies may have a beneficial effect in the prevention and control of AMD and warrant further investigation.

## Data Availability

The data used to support the findings of this study are included within the article and can be available from the corresponding author upon request.
